# Study of Factors Influencing the Oral Bioaccessibility of Commonly Used and Detected Pesticides in Bananas and Mangoes Based on *in vitro* Methods

**DOI:** 10.3390/foods13132019

**Published:** 2024-06-26

**Authors:** Chen Ma, Qun Zhang, Dai-Zhu Lv, Jia Song, Qiong Fan, Hai Tian, Ming-Yue Wang

**Affiliations:** 1 Analysis and Testing Center, Chinese Academy of Tropical Agriculture Sciences, Haikou 571101, China; mc19860112@163.com (C.M.); zhangqun123@zju.edu.cn (Q.Z.); ldz162000@126.com (D.-Z.L.); jia668837@163.com (J.S.); joanhee@126.com (Q.F.); tianhai666@163.com (H.T.); 2 Key Laboratory of Quality and Safety Control for Subtropical Fruit and Vegetable, Ministry of Agriculture and Rural Affairs, Haikou 571101, China; 3 Key Laboratory of Nutritional Quality and Health Benefits of Tropical Agricultural Products of Haikou City, Haikou 571101, China; 4 Hainan Provincial Key Laboratory of Quality and Safety for Tropical Fruits and Vegetables, Haikou 571101, China

**Keywords:** bioaccessibility, influencing factors, pesticides, tropical fruits, *in vitro* methods

## Abstract

Estimating the impact of pesticide residue bioaccessibility in fruits on dietary exposure is a complex task in human health risk assessment. This research investigated the bioaccessibility of ten commonly used and detected pesticides in bananas and mangoes, as well as the factors influencing it, using an *in vitro* model. The highest bioaccessibility was observed at pH levels of 2.5 and 6.5 in the gastric and intestinal stages, respectively. Bioaccessibility decreased significantly with increasing solid/liquid ratios for most pesticides. The consumption of protein and four dietary components (carbohydrates, protein, lipids, and dietary fiber) could significantly reduce pesticide bioaccessibility by 9.89–48.32% (*p* < 0.05). Bioaccessibility in oral and gastric stages among four populations followed the order of adults/the elderly > children > infants, due to decreasing concentrations of α-amylase and pepsin. Pesticides in bananas generally exhibited a higher bioaccessibility (18.65–82.97%) compared to that in mangoes (11.68–87.57%). Bioaccessibility showed a negative correlation with the Log P values of the target pesticide, while no clear relationship was found between bioaccessibility and initial pesticide concentrations. Incorporating bioaccessible pesticide concentrations into risk assessments could lower dietary risk estimates by 11.85–79.57%. Assessing human exposure to pesticides based on bioaccessibility would greatly improve the accuracy of the risk assessment.

## 1. Introduction

Tropical fruits are well known for their high content of bioactive compounds and health-promoting properties, which are attributed to their nutritional composition [1,2]. In China, bananas and mangoes are the most popular and widely consumed tropical fruits. Pesticides are widely used worldwide for crop disease control and prevention [3,4]. Our previous study revealed that approximately 93% and 65% of mango and banana samples contained one or more pesticide residues (mango data not published) [3]. Exposure to pesticide residues is a global concern due to their widespread occurrence and potential risks to human health [5,6,7]. Cooking procedures could decrease pesticide residue levels [8]. Therefore, the dietary intake of pesticide residues through fruits could result in higher exposure because they are frequently consumed raw without cooking [5,9].

To have a negative effect on human health, pesticides must be bioavailable during the digestion process, which depends on the level of bioaccessibility [10,11]. Studies on bioaccessibility have shown that not all pesticides in food can be released from the matrix to digestive fluid, be absorbed by the intestine, and ultimately enter the circulatory system [7,9,10,11]. However, many studies on pesticide exposure in humans assume that all concentrations will be released from food, leading to an overestimation of exposure and health risks [3,5,6]. Therefore, it is crucial to consider bioaccessibility in the dietary risk assessments of pesticide residues in fruits to improve the accuracy of predicting human health risks [12,13]. 

*In vivo* models are widely accepted as the preferred method for evaluating pesticide bioaccessibility, but *in vitro* methods are commonly used as a more cost-effective and efficient alternative [12,13,14]. Bioaccessibility has been extensively studied in the environmental field, particularly concerning human exposure to heavy metals, persistent organic pollutants (POPs), semi-volatile organic compounds (SVOCs), and mycotoxins [8,14,15,16,17,18]. Various factors have been found to influence bioaccessibility, such as digestion conditions (e.g., digestion time, pH, the solid/liquid (S/L) ratio, and concentrations of digestive enzymes) [12,13,15], *in vitro* digestion models [12], food matrices [8,9,15], dietary components [9,12,16], processing methods [18], and properties and concentrations of contaminants [9,14]. Despite a great deal of attention having been paid to pesticide bioaccessibility [7,10,11,12,13], research on the oral bioaccessibility of pesticides during human digestion was quite scarce due to the complexity of pesticide mixtures and the diversity of the food matrix. To our knowledge, the bioaccessibility of pesticide residues and the factors influencing it in banana and mango fruits have not been comprehensively characterized. 

An understanding of bioaccessibility and the influencing factors can provide information for designing effective strategies to protect humans from the exposure risks of pesticides [12,15,16]. This study utilized an *in vitro* model (INFOGEST protocol) [19] that simulates human oral, gastric, and intestinal stages to quantify the bioaccessibility of commonly used and detected pesticide residues in bananas and mangoes in China [3]. The research also explored various factors influencing bioaccessibility, such as digestion conditions (pH, S/L ratio, and digestion time), dietary components (carbohydrates, protein, lipids, and dietary fiber), digestion fluids in four populations (infants, 0–12 months, children, 13–36 months, adults, and the elderly), food matrices (bananas and mangoes), and initial pesticide concentrations (spiked or commercial samples). In addition, human exposure to pesticides was calculated using the bioaccessible concentrations to provide a more realistic estimate of potential health risks. 

## 2. Materials and Methods 

### 2.1. Chemicals and Reagents

Pesticide standards (>99%, purity) (imidacloprid, thiamethoxam, acetamiprid, clothianidin, pyraclostrobin, difenoconazole, carbendazim, tebuconazole, imazalil, and fluxapyroxad) were purchased from the Environmental Quality Supervision and Testing Center, the Ministry of Agriculture, Tianjin, China. The individual stock standard solutions of each pesticide were prepared in methanol and stored at −20 °C in the dark. Matrix-matched mixed working solutions at different concentrations were prepared daily by diluting aliquots of the stock solution using extracts from a blank sample and were stored at 4 °C. 

HPLC-grade acetonitrile, methanol, and formic acid were obtained from Thermo Fisher Scientific (Waltham, MA, USA). For residue analysis, NaCl was purchased from Xilong Chemical Co., Ltd. (Shantou, China). QuEChERS Dispersive SPE Kits (containing 50 mg PSA and 150 mg MgSO_4_) were supplied by Agela Technologies (Tianjin, China).

The simulated oral, gastric, and intestinal fluid reagents were obtained from Xiaodong Yijian (Suzhou, China) Instrument and Equipment Co., Ltd. Dietary components of peanut oil (100 g lipid/100 g), corn starch (100 g carbohydrate/100 g), protein powder (80 g protein/100 g), and dietary fiber powder (85 g fiber/100 g) were purchased from a local supermarket.

Bananas and mangoes were purchased from local markets in Haikou City, China. These fruits are generally consumed after peeling, and pesticide residues present in the pulp could potentially pose risks to human health. In addition, systemic pesticides can penetrate the inner pulp, while non-systemic pesticides remain only in the peels. The protective nature of the peels resulted in very low concentrations of pesticide residues detected in the pulp [3]. To ensure that the fruit pulps contained appropriate levels of pesticide residues, blank samples (without the target pesticide) were peeled and spiked with test pesticide standard working solutions. The spiked samples were allowed to stand for a few hours to allow the spiking solutions to permeate the matrix. The average concentrations of the spiked samples ranged from 826.90 to 1096.40 μg/kg. Both the spiked samples (banana and mango) and commercial mango samples were homogenized and stored at −20 °C until further analysis. 

### 2.2. In vitro Digestion Model

The standardized *in vitro* digestion simulation protocol conducted in this study was based on the INFOGEST protocol, with some modifications [19]. The protocol consisted of three sequential digestive phases: oral, gastric, and intestine. The simulated digestion fluids comprised KCl, KH_2_PO_4_, NaHCO_3_, NaCl, MgCl_2_(H_2_O)_6_, (NH_4_)_2_CO_3_, CaCl_2_(H_2_O)_2_, digestive enzymes, and the concentrations of electrolytes are detailed in Table S1. For the experiment on the “Effects of digestion fluids in four populations on bioaccessibility”, simulated digestion fluids of the adult system were utilized. 

Oral phase. Firstly, the sample (0.5 g) was added in triplicate to 10 mL of SSF (simulated salivary fluid) along with α-amylase (150 U/mL). The pH was adjusted to 7.0 ± 0.1 using 1 mol/L HCl, and the solutions were stirred in a shaking water bath at 37 °C (40 rpm) for 5 min. 

Gastric phase. Subsequently, the pH of the oral fluid was adjusted to 2.5 ± 0.1, and 10 mL of oral digest was mixed with 15 mL of SGF (simulated gastric fluid) containing pepsin (4000 U/mL), then maintained in a shaking water bath at 37 °C (150 rpm) for 2 h. 

Intestinal phase. After the gastric phase, the digestive fluid was adjusted to pH 7.0 ± 0.1 using NaHCO_3_ (1 mol/L), and then 15 mL of SIF (simulated intestinal fluid) containing bile salt (20 mmol/L) and pancreatin (4000 U/mL) was added. The digests were shaken at 150 rpm and incubated at 37 °C for 4 h. After incubation, triplicate samples from each fluid treatment were collected and analyzed for individual pesticides.

### 2.3. Influencing Factors 

#### 2.3.1. Digestion Conditions 

To explore the influence of pesticide bioaccessibility in fruits, the roles of digestion time, pH, and S/L ratio were investigated. The gastrointestinal pH was examined at 1.5, 2.0, 2.5, 3.0, 4.0 and at 6.0, 6.5, 7.0, 7.5, 8.0 during gastric and intestinal digestion, respectively. Digestion times of 1.0, 2.0, 2.5, 3.0, 4.0 h and 1.0, 2.0, 3.0, 4.0, 5.0 h were also studied in the gastric and intestinal stages, respectively. The S/L ratio represents the ratio of food matrix to simulated digestive juice. The S/L ratio was maintained by using varying amounts of fruit samples (0.5, 1.0, 2.0, 3.0, and 5.0 g) with a constant volume of 40 mL digestive juice. All experiments were conducted independently three times. 

#### 2.3.2. Dietary Components 

In the human consumption of fruits, other foods may be ingested successively and mixed in the gastrointestinal environment and affect the bioaccessibility of pesticide residues in fruits. Following Chinese dietary reference intakes (DRIs), and considering the daily secretion of gastrointestinal fluid by an adult (6–8 L) [12,20], carbohydrates (30 g/L), protein (15 g/L), lipids (10 g/L), and dietary fiber (5 g/L) were incorporated into fruit samples to evaluate the influence of dietary components on bioaccessibility. The study contained five treatments (addition of carbohydrates, protein, lipids, dietary fiber, or all four together) and one control group (without dietary components), with each treatment replicated three times.

#### 2.3.3. Digestion Fluids in Four Populations 

Simulated digestion fluids (oral and gastric) of four different age groups were used in this study, including infants (0–12 months), children (13–36 months), adults, and the elderly. Varying concentrations of digestive enzymes were involved in digestion fluids based on the methodology proposed by Xiaodong Yijian (Suzhou, China) Instrument Equipment Co., Ltd. The activity levels of α-amylase in the simulated salivary fluid (SSF) were 15 U/mL for the infant, 50 U/mL for the child, 150 U/mL for the adult, and 200 U/mL for the elderly conditions. In the simulated gastric fluid (SGF), the activity levels of pepsin and lipase were 400 U/mL and 120 U/mL for the infant, 1200 U/mL and 120 U/mL for the child, 4000 U/mL, and 0 U/mL for the adult, and 3000 U/mL and 0 U/mL for the elderly conditions, respectively

#### 2.3.4. Food Matrix and Initial Pesticide Concentrations 

The impact of the food matrix (bananas and mangoes) on pesticide bioaccessibility was investigated using spiked samples with equivalent initial pesticide concentrations. 

To evaluate the influence of initial pesticide concentrations on bioaccessibility, spiked mango samples (with high initial concentrations) and commercial mango samples (with low initial concentrations) were prepared following the banana method.

### 2.4. Chemical Analyses

The extraction procedure for pesticide residues was based on the QuEChERS methodology. After each digestion step, supernatants were separated by centrifugation at 4500× *g* (Centrifuge 5804R, Eppendorf, Hamburg, Germany) for 10 min at 4 °C. Initially, 5 mL of supernatant or 5 g of fruits was extracted with 10 mL of acetonitrile, vigorously shaken for 10 min, and then 5 g of NaCl was added. After centrifugation for 5 min at 4500× *g*, an aliquot of 1 mL of the extract was transferred to 2 mL QuEChERS Dispersive SPE Kits. The extract was vigorously shaken on a vortex shaker for 1 min and then centrifuged at 4500× *g* for 5 min. An aliquot of 1 mL supernatant was diluted in a 1:1 ratio with acetonitrile, filtered through a PTFE 0.22 μm filter, and used for instrumental analysis. 

Pesticide residues were analyzed using a Waters ACQUITY ultraperformance liquid chromatograph coupled with the Xevo TQ-S Triple Quad mass spectrometry system (UPLC-MS/MS) (Waters Co., Milford, MA, USA). Chromatographic separation was achieved on the ACQUITY UPLC BEH C18 column (50 mm × 2.1 mm; 1.7 mm) (Waters, USA) maintained at 35 °C using gradient elution. The mobile phase B was an aqueous solution containing 0.1% formic acid and 5 mmol/L of ammonium acetate, and the mobile phase A was acetonitrile. The gradient was initiated at 10% eluant A for 0–1 min, gradually increased to 70% from 2 to 4 min, further increased to 95% from 6.5 to 9.4 min, and finally decreased to 10% for 0.6 min. The flow rate was 0.30 mL/min, with an injection volume of 5.0 μL. Detection was carried out in the multiple reaction monitoring (MRM) mode, equipped with an electrospray ionization source (ESI^+^). The parameters were as follows: source temperature, 150 °C; capillary voltage, 3.00 kV; cone voltage, 30 V; desolvation gas flow, 800 L/h; cone gas flow, 50 L/h; desolvation temperature, 500 °C. Instrumental parameters were optimized for maximum sensitivity, and the specific MS/MS parameters for the target pesticide are presented in Table S2. Log P values of the target pesticide were obtained from USEPA ECOTOX Database [21], shown in Table S2. 

### 2.5. Method Accuracy

Pesticide concentrations were quantified using the external standard method. The linearity range was assessed at five calibration levels (0.005, 0.010, 0.015, 0.025, and 0.05 mg/mL) in both acetonitrile and matrix (extracted blank fruits and digestion solutions). The calibration exhibited satisfactory linearity, with *R*^2^ values ranging from 0.9920 to 0.9999. Recovery was determined by analyzing blank fruits or digestion solutions fortified at three concentration levels (0.005, 0.01, and 0.1 mg/kg) in five replicates. The recovery rate for each pesticide fell within the range of 76.50–117.33% (Table S3). The limit of quantification (LOQ) was determined as the lowest spiked concentration meeting the recovery criteria, and LOQs for all detected pesticides were 0.005 mg/kg. 

### 2.6. Data Analysis

Data are presented as means ± standard deviations (SD). Statistical analysis was conducted using analysis of variance (ANOVA) followed by the Duncan test at the 0.05 significance level in IBM SPSS Statistics 26.0 (SPSS Inc, Chicago, IL, USA). Curve fittings were simulated in Excel 2013. All figures were drawn using Origin 8.0 (Origin Lab Corporation, Northampton, MA, USA).

The bioaccessibility of pesticides at oral, gastric, and intestinal phases was calculated using the following formula:

Bioaccessibility (%) = C_1_V/C_2_M × 100% (1)

where C_1_ is the concentration of test pesticides in digestion juice (mg/mL), V is the volume of digestion juice (mL), C_2_ is the concentration of test pesticides in the initial fruit sample (mg/kg), and M is the weight of the sample (kg).

The following deterministic approach was carried out to estimate the chronic and acute hazard quotients of pesticides via mango consumption for the general population:

(2) $$\mathrm{cHQ}=\frac{\mathrm{EDI}}{\mathrm{ADI}}\times 100\%=\frac{C\times F}{\mathrm{bw}\times \mathrm{ADI}}\times 100\%$$

(3) $$\mathrm{aHQ}=\frac{\mathrm{EDI}}{\mathrm{ARfD}}\times 100\%=\frac{\mathrm{LP}\times C\times v}{\mathrm{bw}\times \mathrm{ARfD}}\times 100\%$$

where cHQ is the chronic hazard quotient, EDI is the estimated daily intake, ADI is the acceptable daily intake of the detectable pesticide (mg/kg bw), using the values from National Food Safety Standard GB 2763-2021 [22], C is the detected concentration or bioaccessible concentration of pesticide residues in fruits (mg/kg), F is the average daily consumption of mango in China (0.6899 g/kg(bw)/day) [23], bw is average weight of the general population in China (60 kg) [23], aHQ is the acute hazard quotient, ARfD is the acute reference dose of detectable pesticide (mg/kg bw), using the values from the JMPR [24], LP is the consumption of a large meal covering the mango intake of 97.5% of consumers (2.287 g/kg(bw)/day) [23], v is a variability factor representing the ratio of the 97.5th percentile residue to the mean residue in single units (v = 3) [5]. The values of ADI and ARfD of the target pesticides are shown in Table S4.

## 3. Results and Discussion

### 3.1. Effect of Digestion Conditions on Bioaccessibility 

**Figure 1:**
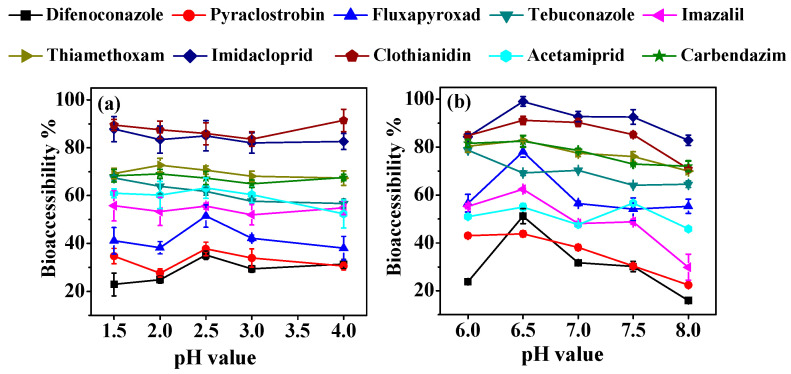
Effect of pH on the bioaccessibility of test pesticides in the gastric (**a**) and intestinal phases (**b**).

In this study, the impact of pH, digestion time, and the S/L ratio on pesticide bioaccessibility was investigated in both gastric and intestinal stages. The influence of gastrointestinal pH on pesticide bioaccessibility is shown in Figure 1. Under the gastric pH conditions (Figure 1a), the bioaccessibility of clothianidin and tebuconazole initially decreased and then slightly dropped (tebuconazole) or increased (clothianidin). Thiamethoxam and carbendazim showed an initial increase in bioaccessibility at pH 1.5–2.0, while other pesticides exhibited a similar trend at pH 2.0–2.5, followed by a gradual decrease with increasing gastric pH. The release of pesticide residue during gastric digestion was relatively higher at pH 2.5, except for thiamethoxam and carbendazim (higher at pH 2.0) and imidacloprid and tebuconazole (higher at pH 1.5). In the simulated intestinal fluid (Figure 1b), the bioaccessibility of most pesticides initially increased, with this followed by a slight or notable drop, except for tebuconazole and acetamiprid. The majority of pesticides showed relatively higher bioaccessibility at pH 6.5 in the simulated intestinal fluid. The variations in pesticide bioaccessibility may be attributed to differences in pesticide stability under varying pH conditions and their affinity for pepsin and pancreatin in the gastric and intestinal phases [13,25,26]. Consequently, pHs 2.5 and 6.5 were recommended as the gastric and intestinal pH values for subsequent experiments.

**Figure 2:**
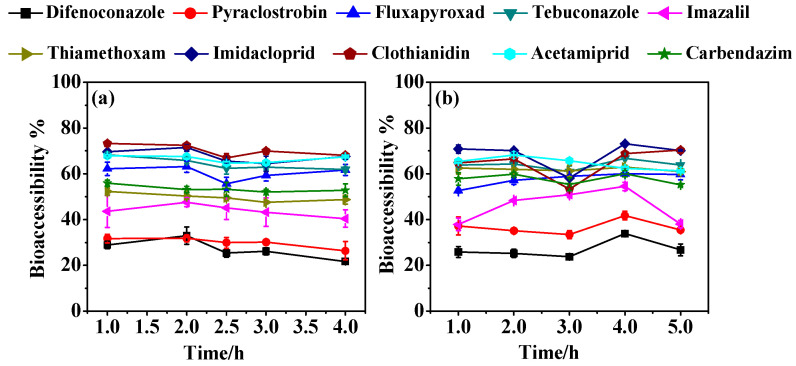
Effect of digestion time on the bioaccessibility of test pesticides in the gastric (**a**) and intestinal phases (**b**).

The impact of digestion time on pesticide bioaccessibility is illustrated in Figure 2. In the gastric phase, most pesticides exhibited an initial increase in bioaccessibility at 1.0–2.0 h, followed by a steady slowing or fluctuating trend at 2.0–4.0 h (Figure 2a). The peak absorption of pesticides was observed at 2.0 h in the gastric phase for most pesticides. In the intestinal phase, pesticide bioaccessibility fluctuated over 1.0–5.0 h and reached the highest absorption values at 4.0 h (Figure 2b). In summary, digestion time influenced the absorption efficiency without significantly changing bioaccessibility. Therefore, incubation times of 2.0 h and 4.0 h were selected for the gastric and intestinal phases in subsequent experiments.

**Figure 3:**
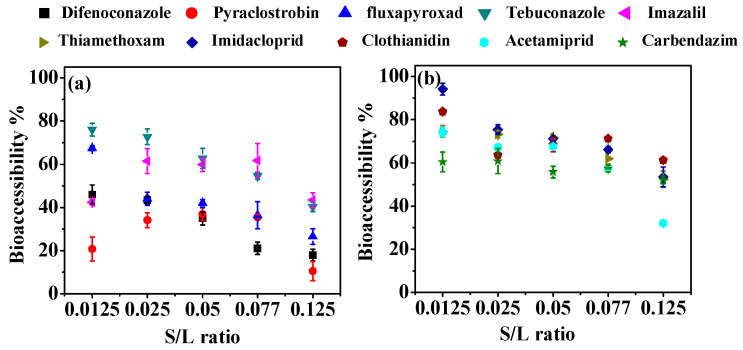
Effect of the S/L ratio on the bioaccessibility of test pesticides in the gastric (**a**) and intestinal phases (**b**).

The S/L ratio, representing the food matrix volume compared to the digestive fluid volume, greatly affects the bioaccessibility of compounds [13,25,26]. As shown in Figure 3, pesticide bioaccessibility was found to be significantly reduced with an increasing S/L ratio from 0.0125 (1/80) to 0.125 (1/8), except for pyraclostrobin and imazalil. The bioaccessibility of these two pesticides showed an initial increase at the S/L ratio of 0.0125 (1/80)–0.025 (1/40) and reached a steady state at 0.025 (1/40)–0.077 (1/13), and this was followed by a notable decrease at 0.077 (1/13)–0.125 (1/8). Furthermore, curve fittings revealed the relationship between *in vitro* bioaccessibility and S/L ratio (Table 1). Fluxapyroxad and imidacloprid displayed a strong logarithmic relationship, with *R*^2^ values of 0.9036 and 0.9438, respectively. For difenoconazole and carbendazim, the curve fittings showed an obvious exponential relationship, with *R*^2^ values of 0.9309 and 0.8520, respectively. Tebuconazole, thiamethoxam, and acetamiprid exhibited a strong linear relationship (*R*^2^ = 0.9970, 0.9571, and 0.9242, respectively). Clothianidin, pyraclostrobin, and imazalil demonstrated a polynomial relationship, with *R*^2^ values of 0.8237, 0.9549, and 0.7843, respectively. As a result, the S/L ratio of 1/80 (0.5 g food matrix) was chosen for the following *in vitro* bioaccessibility tests. Previous studies in apples have shown a strong negative relationship (logarithmic) between bioaccessibility (five pyrethroids, hexaconazole, spirodiclofen, and difenoconazole) and S/L ratio (1/20 to 3/4) [13,25]. Our results were generally consistent with the above literature. In contrast, the release of six pesticides followed an S-curve pattern, which increased with the S/L ratio from 1/500 to 1/10 and stabilized at 1/20 in *Chaenomelis speciosa* [26]. Similar results were observed for pyraclostrobin and imazalil in our study. The discrepancy may be attributed to the differences in pesticide properties and food matrices, and additional research is needed.

### 3.2. Effect of Dietary Components on Bioaccessibility 

**Figure 4:**
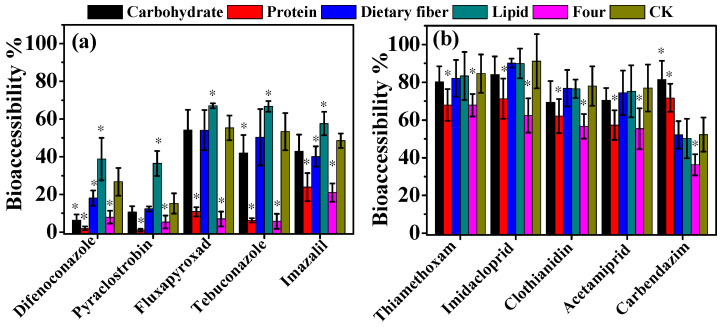
Effects of carbohydrates, protein, lipids, and dietary fiber on the bioaccessibility of tested pesticides, difenoconazole, pyraclostrobin, fluxapyroxad, tebuconazole and imazalil (**a**) thiamethoxam, imidacloprid, acetamiprid, clothianidin and carbendazim (**b**). Stars at the top of columns represent significant differences in bioaccessibility between controls (CK) and tests with the addition of dietary components at a *p*-value of 0.05.

The effects of dietary components (carbohydrate, protein, lipid, and dietary fiber) on bioaccessibility are shown in Figure 4. The results indicated that the addition of protein, dietary fiber, carbohydrates, and all four components together could moderately decrease pesticide bioaccessibility in banana samples by 0.99–48.32%, except for carbendazim. A statistically significant change was observed in the presence of protein and all four components, with decreases of 14.14–47.11% and 9.89–48.32% (*p* < 0.05), respectively. When dietary fiber was added, the bioaccessibility of difenoconazole and imazalil was significantly reduced by 8.62% and 8.39% (*p* < 0.05), respectively. Similarly, the presence of carbohydrates led to a significant reduction in the bioaccessibility of difenoconazole and tebuconazole by 20.41% and 11.40% (*p* < 0.05), respectively. Conversely, protein and carbohydrates significantly increased carbendazim's bioaccessibility by 19.52% and 29.22% (*p* < 0.05), respectively. Lipids were found to significantly enhance the bioaccessibility of more hydrophobic pesticides (difenoconazole, pyraclostrobin, fluxapyroxad, tebuconazole, and imazalil) by 9.07–21.22% (*p* < 0.05), while slightly decreasing the bioaccessibility of other pesticides by 1.22–2.03%.

Dietary components have an important influence on pesticide bioaccessibility. The presence of a complex food matrix during digestion significantly decreased the bioaccessibility of hydrophobic organic compounds compared to digestion in a fasted state [15,27]. It was concluded that the addition of a nutrient matrix led to a reduction in pesticide bioaccessibility, which can be explained by the potential interactions between proteins, complex carbohydrates, and lipids present in the matrix [28]. For instance, bisphenol S's bioaccessibility was reduced by up to 5% after the oral intake of a soy drink [15]. A similar result was observed when mimicking feeding conditions by adding the four dietary components in our study. 

Lipids played a key role in the release of highly lipophilic chemicals, such as pyrethroids, chlorpyrifos, parathion, PBDEs, DDT, PAHs, and PFOA [29,30,31,32]. Lipids facilitated micellarization and chylomicron formation, resulting in a higher bioaccessibility observed in dust and soil samples [29,30,31,32]. However, triazolone's bioaccessibility in cherry tomatoes during the gastrointestinal stage decreased significantly with the addition of trace amounts of oil (0.2 mL), while adding more than 0.4 mL of oil did not further affect bioaccessibility [33]. Bendiocarb, with relatively high water solubility (Log P = 1.7), did not depend on the oil content of the nanoemulsions for bioaccessibility, whereas more hydrophobic pesticides like parathion and chlorpyrifos (Log P = 3.8 and 5.3) exhibited increased bioaccessibility with higher oil content [32,34]. In our study, lipids increased the bioaccessibility of more hydrophobic pesticides (Log P 3.34–4.92), while decreasing the bioaccessibility of other pesticides with higher water solubility (Log P −1.16–2.1) (Table S2). The results probably depend on the polarity of the pesticide molecules. A high lipid content in the digestion solution can facilitate the release of lipophilic chemicals, but it may prevent the migration of pesticides with higher water solubility, thus potentially leading to lower or higher bioaccessibility [32,33,34]. 

Previous literature has reported that carbohydrates (starch) could enhance the bioaccessibility of pyrethroids, PAHs, and PCBs in dust or soil samples, as well as the PBDEs in fish samples [16,35]. In contrast, other findings have indicated that sugar can enhance the overall stability of neonicotinoids in fruit and vegetable powders, leading to a relative reduction in pesticide release [9]. Additionally, it has been observed that carbohydrates may bind to BHT (2,6-di-tert-butyl-hydroxytoluene) to reduce its free concentration and decrease its bioaccessibility by Caco-2 cells [20]. Our results demonstrated that the addition of carbohydrates only increased the bioaccessibility of carbendazim, while having negative effects on the bioaccessibility of other pesticides. 

Uncertain effects on the bioaccessibility of organic chemicals have been observed with the addition of protein [9,16,33,35,36,37]. Yu et al. and Liu et al. found that protein in plant-based foods could negatively affect PBDE's and triazolone's bioaccessibility [33,37]. However, protein could enhance the solubility and desorption of pyrethroids [35], tebuconazole [12], PCB [36], and PBDE [16], and ultimately increase their bioaccessibility. Other reports indicated that the addition of protein significantly increased imidacloprid's bioaccessibility in cucumber, but it decreased the bioaccessibility of thiamethoxam, acetamiprid, and thiacloprid [9]. In our study, protein significantly decreased the bioaccessibility of most target pesticides, except for carbendazim. Proteins are believed to interact with the bioactivities of food and undergo hydrolysis into amino acids, which can influence their release from the food matrix and impact the solubility of chemicals in the digestion solution [9,14,35,37]. This factor warrants further investigation. 

Many studies have suggested that dietary fiber could play a positive role in decreasing the bioaccessibility of neonicotinoids [9], tebuconazole [12], triazolone [33], and PBDE [37]. Our results are in agreement with the previous findings. Dietary fiber, primarily consisting of insoluble and indigestible cellulose, has the ability to capture antinutritional factors, such as polyphenols, within its structure. This leads to the adsorption of chemicals onto the undigested substance, ultimately reducing their bioaccessibility [9,14]. Additionally, dietary fiber can bind with bile acids and phospholipids, inhibiting the formation of micelles and further reducing bioaccessibility [14].

### 3.3. Effect of Digestion Fluids in Different Populations on Bioaccessibility 

This study investigated the effects of simulated digestion fluids in four populations on pesticide bioaccessibility. The oral and gastric digestion fluids with varying concentrations of α-amylase, pepsin, and lipase were detailed in Section 2, Materials and Methods. 

**Figure 5:**
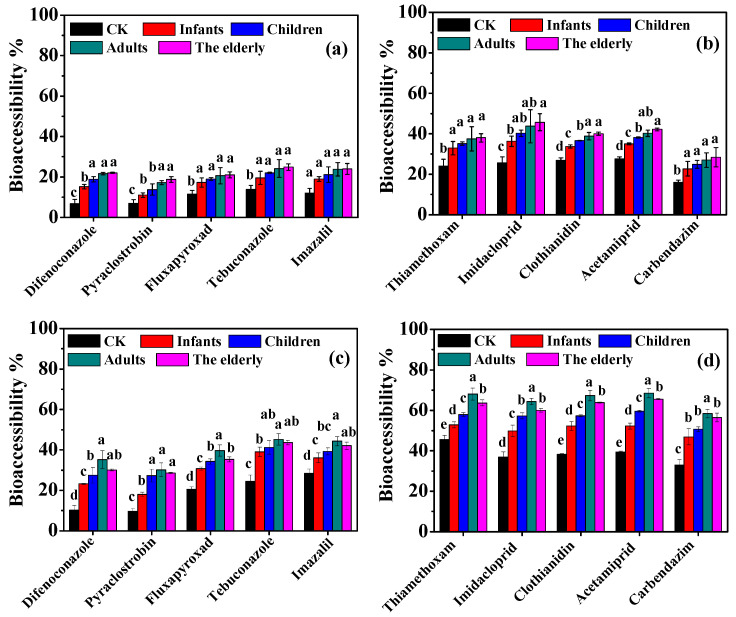
Effect of simulated digestion fluids in four populations (infants, 0–12 months, children, 13–36 months, adults, and the elderly) on the bioaccessibility of tested pesticides in the oral (**a**,**b**) and gastric (**c**,**d**) phases. CK is the control without digestive enzymes. Bars with different lowercase letters are significantly different at a *p*-value of 0.05.

Figure 5a,b demonstrate that bioaccessibility in the oral stage followed the order CK < infants < children < adults < the elderly, which clearly showed a gradually increasing effect of α-amylase on bioaccessibility across the four populations. A significant increase in bioaccessibility was observed when comparing controls without digestive enzymes to samples in simulated digestion fluids for the four populations (*p* < 0.05). The increase in bioaccessibility ranged from 4.17 to 10.69%, 6.90 to 14.65%, 9.07 to 18.17%, and 9.50 to 20.13% for the infant, child, adult, and elderly conditions, respectively. Under the child, adult, and elderly digestion conditions, the bioaccessibility of difenoconazole, clothianidin, and acetamiprid was significantly higher than that in the infant digestion condition (*p* < 0.05). Pyraclostrobin's bioaccessibility was significantly higher in the adult and elderly conditions compared to that in the infant and child conditions (*p* < 0.05). Additionally, imidacloprid's bioaccessibility in the elderly group was significantly higher than it was in the infant group (*p* < 0.05). Xiao et al. discovered that the presence of α-amylase enhanced the bioaccessibility of six pesticides (chlorpyrifos, phoxim, imidacloprid, thiamethoxam, fenpropathrin, and emamectin benzoate) in *Chaenomelis speciosa* [26]. This increase may be attributed to the function of α-amylase to break down carbohydrates (starch) in food, thus reducing the trapping or binding of pesticides [14]. Our results in this study were consistent with previous studies [26]. 

In the gastric stage, the highest bioaccessibility was observed in adult digestion (30.17–68.55%), followed by that in the elderly (28.57–65.57%), children (27.34–59.57%), and infants (18.02–52.89%) (Figure 5c,d). In comparison with gastric fluids without digestive enzymes, bioaccessibility was significantly enhanced by 1.16–2.26 times, 1.27–2.83 times, 1.40–3.42 times, and 1.40–2.96 times for the infant, child, elderly, and adult conditions, respectively. Bioaccessibility increased with the increasing concentrations of pepsin in the gastric digestion fluids of the four populations. Significant changes in bioaccessibility were observed for thiamethoxam, acetamiprid, and clothianidin among the gastric digestion fluids of the four populations (*p* < 0.05). For other pesticides, bioaccessibility in the infant condition was significantly lower than that in the adult and the elderly conditions (*p* < 0.05). Xiao et al. found that pepsin increased the bioaccessibility of chlorpyrifos, phoxim, imidacloprid, and fenpropathrin at concentrations of 0.00–0.10% and decreased at concentrations of 0.10–0.20% [26]. Similar results have shown that pepsin led to a slight increase in the liberation of halogenated flame retardants, organophosphorus flame retardants, and polycyclic aromatic hydrocarbons from particles [38]. However, there was no significant difference in bisphenol S's bioaccessibility between the control and the treatments with the addition of enzymes in the oral and gastric phases [15]. Li et al. demonstrated that the total peptides released were significantly lower in the elderly condition with a low pepsin concentration compared to that in adult digestion with a high pepsin concentration [39]. This suggests that pepsin can accelerate protein hydrolysis and enhance the release of pesticides from the food matrix [14,26,38]. On the other hand, pepsin could hydrolyze and metabolize the pesticides [26]. Gastric lipase in the systems of infants and children contributes to the breakdown of dietary fat in the gastric environment, leading to formation of hydrophobic micelles that increase bioaccessibility [14,15]. However, since the food matrix used in this study was banana with an extremely low lipid content, gastric lipase is expected to have a minimal impact on bioaccessibility. Bile salts and pancreatin in the intestinal phase also play crucial roles in influencing the bioaccessibility of chemicals [14,26]. Therefore, further research could be necessary to explore the effects of intestinal digestion fluids in the four populations on bioaccessibility. 

### 3.4. Effect of Food Matrix on Bioaccessibility 

**Figure 6:**
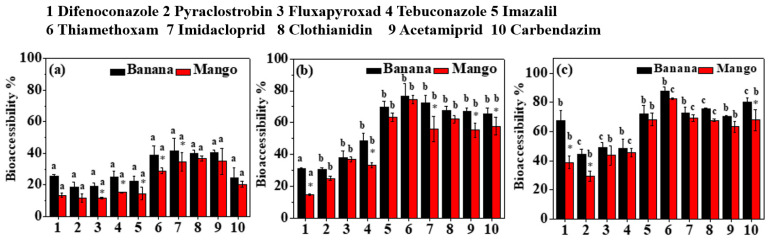
Effect of food matrix (banana and mango) on the bioaccessibility of tested pesticides in the oral (**a**), gastric (**b**) and intestinal (**c**) phase. Stars at the top of columns represent significant differences in bioaccessibility between banana and mango at a *p*-value of 0.05. Bars with different lowercase letters are significantly different in terms of the same pesticide among oral, gastric, and intestinal phases at a *p*-value of 0.05.

Results have shown that food matrices significantly influenced pesticide bioaccessibility. Figure 6 indicates that pesticide bioaccessibility in bananas ranged from 18.65% to 41.49% in the oral phase, 30.75% to 76.61% in the gastric phase, and 44.58% to 87.97% in the intestinal phase, respectively. In mangoes, pesticide bioaccessibility in the oral, gastric, and intestinal phases ranged from 11.62 to 36.85%, 14.65 to 74.42%, and 29.55 to 82.57%, respectively. The bioaccessibility of the target pesticides was significantly lower in the oral phase compared to that in the gastric and intestinal phases (*p* < 0.05), except for difenoconazole. Furthermore, certain pesticides exhibited significantly lower bioaccessibility in the gastric phase compared to that in the intestinal phase (*p* < 0.05), including difenoconazole, clothianidin, pyraclostrobin, fluxapyroxad, and carbendazim in banana samples and difenoconazole, clothianidin, tebuconazole, and thiamethoxam in mango samples. Overall, all the tested pesticides presented higher bioaccessibility in bananas compared to that in mangoes. Additionally, significant differences in bioaccessibility were observed for specific pesticides during the three phases (*p* < 0.05). This study suggests that a daily intake of the target pesticides from mangoes may be relatively safer than that from bananas.

The differential sorption of chemicals in matrices may lead to varying solubility and release of certain contaminants. Previous research has demonstrated that pesticide bioaccessibility varied depending on the food matrices [8,9,14,40]. Food composition, such as pectins, cellulose, lipids, proteins, carbohydrates, anthocyanins, and procyanidins, plays a significant role in influencing organic compounds' bioaccessibility [8,17,40,41]. Faria et al. reported that heterocyclic aromatic amines (HAAs) had higher bioaccessibility in a vegetarian diet, while PAHs and mycotoxins (OTA) were more bioaccessible in a Western diet [17]. Moreover, the bioaccessibility of dichlorodiphenyltrichloroethane and its metabolites (DDXs) and hexachlorocyclohexane isomers (HCHs) varied in different food types (meat, vegetables, fruit, cereal, and aquatic food) and the bioaccessibility was positively associated with lipid contents and negatively correlated with insoluble fiber and carbohydrates in foods [41]. Significant differences (*p* < 0.05) in the bioaccessibility of neonicotinoids were observed in tomatoes, cucumbers, and carrots, and the bioaccessibility was negatively correlated with sugars in foods [9]. However, carbohydrates in plant-based food had a positive correlation with PBDE's bioaccessibility [37]. In our study, the higher bioaccessibility observed in bananas could be attributed to the higher carbohydrate content (17.2–30.2 g/100g) in bananas compared to that in mangoes (11.4–18.0 g/100 g) (data not yet published). The influence of carbohydrates in food on bioaccessibility remains controversial. Consequently, further investigation was needed to explore the effects of nutrient contents in foods and the physicochemical properties of pollutants on bioaccessibility.

### 3.5. Effect of Initial Pesticide Concentration on Bioaccessibility 

Table 2 presents the initial and bioaccessible concentrations of the tested pesticides in spiked and commercial mango samples, along with chronic or acute hazard quotients for the general population. The results demonstrate that bioaccessibility varied greatly according to the types of pesticides. Neonicotinoids (imidacloprid, thiamethoxam, clothianidin, and acetamiprid) and carbendazim exhibited relatively higher bioaccessibility, ranging from 57.68 ± 0.24 to 88.13 ± 2.50% and from 54.32 ± 0.54 to 57.91 ± 5.08%, respectively. On the other hand, triazoles (difenoconazole, tebuconazole, imazalil), pyraclostrobin, and fluxapyroxad had lower bioaccessibility values, of 20.29 ± 4.52–54.54 ± 0.11%, 29.55 ± 2.32–40.33 ± 3.37%, and 42.31 ± 0.35–48.88 ± 0.64%, respectively. The bioaccessibility values showed a negative correlation with the Log P values for the target pesticide (Figure S1, *R*^2^ = 0.9029). This result was consistent with previous results [32,41,42]. Some studies have shown that the bioaccessibility of PBDEs, PCBs, and DDTs are not correlated with their initial concentrations [14,43,44]. Similar results were obtained in our study. In contrast, other studies have indicated that contaminant concentrations can affect bioaccessibility. For instance, there was a negative correlation between the initial MeHg concentration in seafood and the bioaccessibility of Hg [45]. Additionally, Cr's bioaccessibility was negatively correlated with Cr contents in mushrooms [18]. Thus, further research could be conducted to study the impact of initial pesticide concentrations on bioaccessibility. 

### 3.6. Dietary Risk Assessment of Pesticides

Numerous studies have demonstrated that integrating bioaccessibility into dietary exposure assessments markedly decreases the estimated exposure dose [8,14,28,40,41,42]. The values of chronic hazard quotients (cHQs) and acute hazard quotients (aHQs) in our study ranged from 0.012% to 0.120% and 0.011% to 0.241% in spiked samples based on the initial pesticide concentration (Table 2). After correction by bioaccessibility, there was a notable decrease of 17.42% to 70.41% in exposure risk for the general population. The HQ values were between 0.0002% and 0.0103% for the cHQ and between 0.0001% and 0.0383% for the aHQ in commercial samples after the consideration of bioaccessibility. Without considering bioaccessibility in commercial samples, HQs for pesticides could be overestimated by 11.85–79.57%. Overall, exposure to pesticide residues in spiked and commercial mango samples was found to be very low and in all cases within the acceptable range. These results were consistent with earlier studies. When considering bioaccessibility, the health risks of pentachlorophenol in animal-derived foods and triazole fungicides in grapes were reduced by 42.70–98.46% and 5.79–27.34%, respectively [8,42]. Thus, the incorporation of bioaccessibility into dietary exposure assessments can greatly improve the accuracy of the risk assessment. 

## 4. Conclusions

This study investigated the bioaccessibility of commonly used and detected pesticides in bananas and mangoes using *in vitro* methods. The research also explored various factors that influence bioaccessibility, such as digestion conditions (pH, S/L ratio, and digestion time), dietary components, digestion fluids in four populations, food matrices, and initial pesticide concentrations. The study found that bioaccessibility reached its relatively highest values at pHs 2.5 and 6.5 in the gastric and intestinal stages, respectively. Bioaccessibility decreased significantly with an increasing S/L ratio, except for pyraclostrobin and imazalil. The addition of protein, dietary fiber, carbohydrates, and the four dietary components led to a moderate reduction in pesticide bioaccessibility, particularly with protein (14.14–47.11% decrease) and the four components (9.89–48.32% decrease) (*p* < 0.05). The bioaccessibility in oral and gastric stages among four populations ranked in the order of adults or the elderly > children > infants due to the decreasing concentrations of α-amylase and pepsin. Pesticides in bananas presented a higher bioaccessibility than that in mangoes. There were no clear correlations between bioaccessibility and initial pesticide concentrations. Bioaccessibility showed a negative correlation with Log P values for the target pesticide. Incorporating bioaccessible pesticide concentrations into risk assessments significantly reduced risk estimates by 11.85–79.57%. The study emphasizes the importance of considering pesticide bioaccessibility in dietary exposure calculations to improve the accuracy of exposure results and enhance the reliability of data used in regulatory decisions.

## Figures and Tables

**Table 1:** Curve fittings between the pesticide bioaccessibility and the S/L ratio along with the corresponding correlation coefficients.

Pesticides	Fitted Equation					
	Linear	*R* ^2^	Exponential	*R* ^2^	Logarithmic	*R* ^2^
Difenoconazole	y = −270.3x + 48.316	0.9130	y = 51.823e^−9.156x^	0.9309	y = −13.34ln(x) − 9.263	0.9155
Fluxapyroxad	y = −292.9x + 60.226	0.7606	y = 61.990e^−7.009x^	0.8703	y = −15.74ln(x) − 6.187	0.9036
Tebuconazole	y = −320.72x + 79.838	0.9970	y = 83.059e^−5.683x^	0.9965	y = −15.18ln(x) + 13.567	0.9191
Thiamethoxam	y = −205.76x + 78.693	0.9571	y = 79.948e^−3.258x^	0.9529	y = −9.181ln(x) + 37.932	0.7840
Imidacloprid	y = −305.38x + 89.74	0.8564	y = 91.195e^−4.358x^	0.9100	y = −15.81ln(x) + 22.395	0.9438
Acetamiprid	y = −236.23x + 53.497	0.9242	y = 57.629e^−7.076x^	0.8852	y = −10.33ln(x) + 7.3547	0.7273
Carbendazim	y = −74.264x + 61.581	0.8486	y = 61.721e^−1.317x^	0.8520	y = −3.503ln(x) + 46.275	0.7768
Clothianidin	Polynomial y = −180618x^3^ + 37621x^2^ − 2220.4x + 103.51 *R*^2^ = 0.8237					
Pyraclostrobin	Polynomial y = −6630.7x^2^ + 794.94x + 14.50 *R*^2^ = 0.9549					
Imazalil	Polynomial y = −8707.1x^2^ + 1140.5x + 57.86 *R*^2^ =0.7843					

**Table 2:** The initial and bioaccessible concentrations of pesticide residues in spiked and commercial mango samples and chronic or acute hazard quotients for the general population.

Pesticides	Initial Concentration (μg/kg)	Bioaccessibility (%)	Bioaccessible Concentration (μg/kg)	CHQ ^a^ (%)	cHQ-BA ^b^ (%)	aHQ ^a^ (%)	aHQ-BA ^b^ (%)
Spiked sample							
Difenoconazole	1040.00 ± 6.32	38.81 ± 4.38	403.81 ± 48.01	1.20 × 10^−1^	4.64 × 10^−2^	3.96 × 10^−2^	1.54 × 10^−2^
Pyraclostrobin	1052.00 ± 24.51	29.55 ± 2.32	311.25 ± 31.65	4.03 × 10^−2^	1.19 × 10^−2^	2.41 × 10^−1^	7.12 × 10^−2^
Fluxapyroxad	826.90 ± 1.18	43.57 ± 4.83	360.32 ± 40.45	4.75 × 10^−2^	2.07 × 10^−2^	3.15 × 10^−2^	1.37 × 10^−2^
Tebuconazole	1092.80 ± 19.97	45.69 ± 3.81	499.81 ± 50.76	4.19 × 10^−2^	1.92 × 10^−2^	4.17 × 10^−2^	1.91 × 10^−2^
Imazalil	931.00 ± 26.56	48.36 ± 4.27	450.99 ± 52.60	3.57 × 10^−2^	1.73 × 10^−2^	2.13 × 10^−1^	1.03 × 10^−1^
Thiamethoxam	1001.13 ± 13.41	82.57 ± 0.69	826.69 ± 17.98	1.44 × 10^−2^	1.19 × 10^−2^	1.14 × 10^−2^	9.45 × 10^−3^
Imidacloprid	962.29 ± 25.88	69.18 ± 2.50	666.14 ± 41.96	1.84 × 10^−2^	1.28 × 10^−2^	2.75 × 10^−2^	1.90 × 10^−2^
Clothianidin	1048.40 ± 6.80	67.74 ± 0.89	710.23 ± 13.94	1.48 × 10^−2^	9.34 × 10^−3^	1.03 × 10^−1^	6.51 × 10^−2^
Acetamiprid	898.30 ± 2.50	63.32 ± 3.86	568.87 ± 36.26	1.21 × 10^−2^	8.17 × 10^−3^	2.00 × 10^−2^	1.35 × 10^−2^
Carbendazim	1096.40 ± 9.00	57.91 ± 5.08	635.23 ± 60.91	4.20 × 10^−2^	2.43 × 10^−2^	1.57 × 10^−1^	9.08 × 10^−2^
Commercial sample 1							
Difenoconazole	93.03 ± 6.33	23.27 ± 0.41	21.67 ± 1.85	1.07 × 10^−2^	2.50 × 10^−3^	3.60 × 10^−3^	8.00 × 10^−4^
Pyraclostrobin	141.05 ± 8.54	31.31 ± 0.89	44.21 ± 3.93	5.40 × 10^−3^	1.70 × 10^−3^	3.23 × 10^−2^	1.01 × 10^−2^
Fluxapyroxad	106.45 ± 4.38	48.88 ± 0.64	52.05 ± 2.82	6.10 × 10^−3^	3.00 × 10^−3^	4.10 × 10^−3^	2.00 × 10^−3^
Tebuconazole	32.89 ± 2.45	54.54 ± 0.11	17.94 ± 1.37	1.30 × 10^−3^	7.00 × 10^−4^	1.30 × 10^−3^	7.00 × 10^−4^
Imidacloprid	19.89 ± 0.91	78.72 ± 0.39	15.66 ± 0.79	4.00 × 10^−4^	3.00 × 10^−4^	6.00 × 10^−4^	5.00 × 10^−4^
Commercial sample 2							
Difenoconazole	180.80 ± 8.44	20.29 ± 4.52	36.94 ± 9.89	2.08 × 10^−2^	4.20 × 10^−3^	6.90 × 10^−3^	1.40 × 10^−3^
Pyraclostrobin	80.48 ± 4.49	40.33 ± 3.37	32.56 ± 4.52	3.10 × 10^−3^	1.20 × 10^−3^	1.84 × 10^−2^	7.40 × 10^−3^
Clothianidin	168.92 ± 2.32	88.13 ± 2.50	148.91 ± 6.27	1.90 × 10^−3^	1.70 × 10^−3^	3.20 × 10^−3^	2.80 × 10^−3^
Commercial sample 3							
Difenoconazole	134.33 ± 16.44	43.12 ± 2.04	58.15 ± 9.83	1.54 × 10^−2^	6.70 × 10^−3^	5.10 × 10^−3^	2.20 × 10^−3^
Pyraclostrobin	284.87 ± 17.89	32.55 ± 1.82	92.94 ± 11.01	1.09 × 10^−2^	3.60 × 10^−3^	6.51 × 10^−2^	2.13 × 10^−2^
Imidacloprid	370.28 ± 16.63	79.06 ± 0.53	292.80 ± 15.11	7.10 × 10^−3^	5.60 × 10^−3^	1.06 × 10^−2^	8.40 × 10^−3^
Carbendazim	135.38 ± 4.53	55.03 ± 0.83	74.52 ± 3.62	5.20 × 10^−3^	2.90 × 10^−3^	1.94 × 10^−2^	1.07 × 10^−2^
Commercial sample 4							
Pyraclostrobin	168.54 ± 1.88	30.76 ± 0.65	51.85 ± 1.67	6.50 × 10^−3^	2.00 × 10^−3^	3.85 × 10^−2^	1.19 × 10^−2^
Fluxapyroxad	140.81 ± 3.75	42.31 ± 0.35	59.59 ± 2.08	8.10 × 10^−3^	3.40 × 10^−3^	5.40 × 10^−3^	2.30 × 10^−3^
Thiamethoxam	17.36 ± 0.70	65.21 ± 1.22	11.33 ± 0.67	2.00 × 10^−4^	2.00 × 10^−4^	2.00 × 10^−4^	1.00 × 10^−4^
Imidacloprid	20.01 ± 0.34	57.68 ± 0.24	11.54 ± 0.24	4.00 × 10^−4^	2.00 × 10^−4^	6.00 × 10^−4^	3.00 × 10^−4^
Clothianidin	132.54 ± 2.58	72.20 ± 0.16	95.70 ± 2.07	1.50 × 10^−3^	1.10 × 10^−3^	2.50 × 10^−3^	1.80 × 10^−3^
Acetamiprid	32.60 ± 0.91	75.28 ± 0.13	24.54 ± 0.73	5.00 × 10^−4^	4.00 × 10^−4^	3.70 × 10^−3^	2.80 × 10^−3^
Carbendazim	493.86 ± 7.24	54.32 ± 0.54	268.29 ± 6.60	1.89 × 10^−2^	1.03 × 10^−2^	7.06 × 10^−2^	3.83 × 10^−2^

^a^ Hazard quotient calculated with the initial concentration of the target pesticide; ^b^ hazard quotient calculated with the bioaccessible concentration of the target pesticide.

## Data Availability

The original contributions presented in the study are included in the article/Supplementary Materials; further inquiries can be directed to the corresponding authors.

## Supplementary Materials

The following supporting information can be downloaded at https://www.mdpi.com/article/10.3390/foods13132019/s1, Table S1. The concentrations of electrolytes in simulated digestion fluids; Table S2. The optimal MS/MS parameters of test pesticides for UHPLC-MS/MS and Log P values; Table S3. Analytical recoveries, relative standard deviations (RSDs), calibration curves of correlation coefficient (*R*^2^) values; Table S4. ADI and ARfD of the target pesticides. Figure S1: Relationships between the bioaccessibility of the target pesticides and their Log P values.
