# Supplementary material for: Study of Factors Influencing the Oral Bioaccessibility of Commonly Used and Detected Pesticides in Bananas and Mangoes Based on in vitro Methods
Source: Foods. 2024 Jun 26;13(13):2019. doi: 10.3390/foods13132019 (PMC11241204; doi:10.3390/foods13132019)
Supplement: Supplementary file 1 [file foods-13-02019-s001.zip › foods-3072424-supplementary.pdf]

Supplementary Materials.

Table S1. The concentrations of electrolytes in simulated digestion fluids.

Constituent	SSF (mmol/L)	SGF (mmol/L)	SIF (mmol/L)
K^+	18.80	7.80	7.60
$H_2PO_4^-$	3.70	0.90	2.80
HCO_3^-, CO_3^{2-}	13.70	25.50	85.00
Cl^-	19.50	70.20	55.50
Mg^{2+}	0.15	0.10	0.33
NH^{4+}	0.12	1.00	-
Na^+	13.60	72.20	123.40
Ca^{2+}	1.50	0.15	0.60

Table S2. The optimal MS/MS parameters of test pesticides for UHPLC-MS/MS and Log P values

Pesticides	Retention time (min)	Parent (m/z)	Daughter (m/z)	Dwell (s)	Cone (V)	Collision (eV)	Log P *
Difenoconazole	3.31	406.1	337.0/251.0	0.020	25	24/35	4.92
Pyraclostrobin	3.53	388.1	194.4/163.1	0.020	50	12/24	4.25
Fluxapyroxad	2.91	388.2	342.1/234.1	0.020	25	20/20	4.76
Tebuconazole	3.02	308.2	70.0/125.0	0.020	57	45/55	3.34
Imazalil	2.44	297.3	158.9/255.0	0.020	25	32/24	3.58
Thiamethoxam	2.14	292.1	211.2/131.7	0.020	30	12/20	-1.16
Imidacloprid	2.28	256.1	175.1/209.1	0.020	34	20/15	-0.43
Acetamiprid	2.32	223.0	125.9/90.0	0.020	27	20/34	0.62
Clothianidin	2.24	250.0	83.2/125.0	0.020	34	28/24	0.4
Carbendazim	1.86	192.0	160.1/132.1	0.020	33	18/28	2.1

* values available from the USEPA ECOTOX Database [21]

Table S3. Analytical average recoveries (AR), relative standard deviations (RSDs), calibration curves of correlation coefficient (R^2) values

Pesticides	Spiked level (mg/kg)	Oral juice			Gastric juice			Intestinal juice			Banana			Mango		
		AR (%)	RSD (%)	R^2	AR (%)	RSD (%)	R^2	AR (%)	RSD (%)	R^2	AR (%)	RSD (%)	R^2	AR (%)	RSD (%)	R^2
Difenoconazole	0.005	86.33	1.38	0.9998	108.25	3.65	0.9986	107.20	2.69	0.9997	90.83	5.50	0.9998	91.76	4.75	0.9994
	0.01	102.67	1.33		119.80	5.45		100.33	1.46		92.34	6.20		97.31	6.05	
	0.1	108.67	1.11		107.33	1.10		115.40	3.16		98.75	8.59		98.16	1.51	
Pyraclostrobin	0.005	94.83	2.91	0.9946	92.83	1.25	0.9995	93.70	1.25	0.9994	103.51	2.05	0.9999	99.84	5.72	0.9997
	0.01	105.00	1.61		103.33	1.72		100.83	1.81		113.09	3.93		95.56	4.77	
	0.1	116.17	2.72		105.00	2.60		99.54	1.22		96.45	5.51		92.49	1.72	
Fluxapyroxad	0.005	89.33	1.56	0.9924	98.54	4.25	0.9920	95.40	1.89	0.9944	98.57	9.99	0.9973	115.00	2.18	0.9935
	0.01	112.70	4.75		105.30	2.25		101.60	3.69		105.06	7.32		106.88	6.46	
	0.1	111.80	1.46		108.90	2.23		114.40	3.23		93.12	10.58		96.46	4.41	
Tebuconazole	0.005	92.00	1.10	0.9993	94.22	1.76	0.9998	104.83	2.15	0.9966	98.21	2.22	0.9996	98.61	5.07	0.9972
	0.01	96.00	1.04		98.22	1.92		103.67	3.81		112.71	6.27		103.26	4.89	
	0.1	95.13	2.24		100.44	3.73		106.17	2.22		101.04	6.05		92.66	3.14	
Imazalil	0.005	78.83	1.55	0.9997	76.50	2.99	0.9997	81.83	2.50	0.9974	89.92	10.80	0.9997	86.10	4.35	0.9998
	0.01	85.00	2.54		82.00	3.54		87.50	1.81		87.19	5.76		88.51	3.67	

	0.1	112.00	3.09		117.33	4.14		98.13	3.10		95.62	2.34		94.61	2.44	
Thiamethoxam	0.005	81.13	2.38	0.9978	86.89	1.86	0.9985	84.91	2.88	0.9964	94.64	3.03	0.9980	98.54	8.47	0.9996
	0.01	95.73	3.25		87.56	5.08		96.00	4.65		94.12	9.10		90.61	1.18	
	0.1	116.67	4.14		93.56	2.56		95.33	3.19		96.14	5.11		97.26	1.60	
Imidacloprid	0.005	85.47	3.16	0.9993	94.57	2.05	0.9984	85.17	1.08	0.9963	90.98	4.44	0.9978	94.44	5.78	0.9998
	0.01	105.62	2.27		102.19	4.14		103.33	5.23		98.44	6.12		98.27	8.57	
	0.1	99.56	4.54		104.00	3.71		82.00	3.43		95.89	1.36		91.50	1.07	
Acetamiprid	0.005	95.33	1.23	0.9994	98.89	4.69	0.9995	109.33	4.96	0.9987	91.32	1.46	0.9994	96.34	1.76	0.9999
	0.01	101.78	2.79		100.89	3.66		115.56	1.11		92.03	1.13		96.58	7.05	
	0.1	100.05	3.85		105.69	1.24		101.78	3.42		90.74	3.09		92.66	3.48	
Clothianidin	0.005	104.67	3.65	0.9995	85.73	4.13	0.9999	107.20	5.22	0.9973	95.58	3.20	0.9994	96.00	5.38	0.9996
	0.01	107.11	2.23		95.11	1.29		104.67	2.08		91.72	4.37		95.53	1.87	
	0.1	107.33	4.45		102.33	2.10		103.50	3.36		91.39	8.57		95.77	3.62	
Carbendazim	0.005	95.33	1.35	0.9995	92.01	2.31	0.9997	97.50	1.45	0.9991	92.52	3.15	0.9991	90.42	5.80	0.9996
	0.01	98.60	4.98		98.70	3.17		99.20	1.51		98.76	3.74		93.14	1.96	
	0.1	114.00	1.51		110.10	4.74		100.50	3.17		93.54	2.27		92.22	2.77	

Table S4. ADI and ARfD of the target pesticides

Pesticides	ADI (mg/kg bw)	ARfD (mg/kg bw)
Difenoconazole	0.01	0.3
Pyraclostrobin	0.03	0.05
Fluxapyroxad	0.02	0.3
Tebuconazole	0.03	0.3
Imazalil	0.03	0.05
Thiamethoxam	0.08	1
Imidacloprid	0.06	0.4
Acetamiprid	0.07	0.1
Clothianidin	0.1	0.6
Carbendazim	0.03	0.08

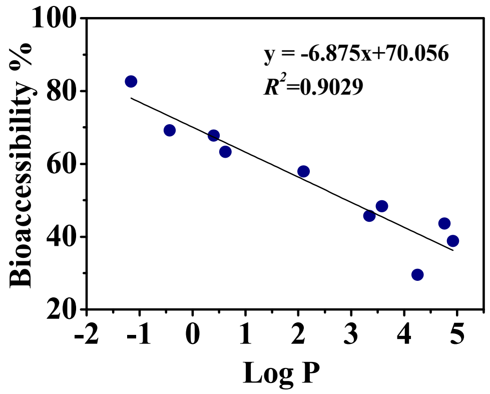

Figure S1. Relationships between the bioaccessibility of the target pesticides and their Log P values.
